# Enhanced Cognition and Modulation of Brain Connectivity in Mild Neurocognitive Disorder: The Promise of Transcranial Pulse Stimulation

**DOI:** 10.3390/biomedicines12092081

**Published:** 2024-09-12

**Authors:** Heidi Ka-Ying Lo, Tommy Kwan-Hin Fong, Teris Cheung, Sze-Ting Joanna Ngan, Wai-Yan Vivian Lui, Wai-Chi Chan, Corine Sau-Man Wong, Teenie Kwan-Tung Wong, Calvin Pak-Wing Cheng

**Affiliations:** 1 Department of Psychiatry, The University of Hong Kong, Hong Kong, China; lokaying@hku.hk (H.K.-Y.L.); u3550211@connect.hku.hk (T.K.-H.F.); joannangan17@gmail.com (S.-T.J.N.); wylui221@hku.hk (W.-Y.V.L.); waicchan@hku.hk (W.-C.C.); teeniew@hku.hk (T.K.-T.W.); 2 School of Nursing, The Hong Kong Polytechnic University, Hong Kong, China; teris.cheung@polyu.edu.hk; 3 Division of Community Medicine and Public Health Practice, The University of Hong Kong, Hong Kong, China; wongcsm@hku.hk

**Keywords:** neurocognitive disorder (NCD), non-invasive brain stimulation (NIBS), transcranial pulse stimulation (TPS), ultrashort ultrasound pulses, functional connectivity (FC), brain connectivity, neurocognitive performance, mild neurocognitive disorder (mNCD), fMRI, default mode network (DMN)

## Abstract

Existing pharmacological treatments for mild neurocognitive disorder (NCD) offer limited effectiveness and adverse side effects. Transcranial pulse stimulation (TPS) utilizing ultrashort ultrasound pulses reaches deep brain regions and may circumvent conductivity issues associated with brain stimulation. This study addresses the gap in TPS research for mild NCD during a critical intervention period before irreversible cognitive degradation. Our objective was to explore the effectiveness and tolerability of TPS in older adults with mild NCD. In an open-label study, 17 older adults (including 10 females and 7 males) with mild NCD underwent TPS for two weeks with three sessions per week. Cognitive evaluations and fMRI scans were conducted pre- and post-intervention. The results indicated changes in functional connectivity in key brain regions, correlating with cognitive improvement at *B* = 0.087 (CI, 0.007–0.167; *p* = 0.038). However, cortical thickness measurements showed no significant differences. Here we show that TPS can enhance cognitive function within mild NCD. This proof-of-concept study suggests that TPS has potential as a non-invasive therapy used to attenuate cognitive decline, encouraging further investigation in larger randomized trials. The findings could influence clinical practice by introducing TPS as an adjunctive treatment option and potentially impact policy by promoting its inclusion in new treatment strategies for mild NCD.

## 1. Introduction

Neurocognitive disorder (NCD), previously known as dementia, is a worldwide health burden characterized by an irreversible trajectory of cognitive decline. Interventions targeting the amelioration of cognitive decline or the prevention of NCD progression are of paramount importance, potentially mitigating the impact of NCD on affected individuals, their families, and society at large [1]. Mild neurocognitive disorder (mNCD) is defined by a noticeable decline in cognitive functioning that is more severe than in normal aging but which does not interfere significantly with daily life activities [2,3,4]. The prevalence of mNCD in older adults is approximately 20.3%; mNCD affects both sexes equally and can involve impairments in various cognitive domains, with learning and memory being the most frequently affected [5]. Eventually, mNCD can progress to major neurocognitive disorders [2,6]. Despite wide-ranging research elucidating the putative biological mechanisms of NCD, the evidence suggests existing pharmacological treatments offer limited effectiveness and potential adverse side effects in preventing, redacting, or postponing cognitive decline during the early stages of NCD [7]. While there is no definitive treatment, ongoing research and non-pharmacological interventions like non-invasive brain stimulation (NIBS) show beneficial effects on the improvement and maintenance of cognitive functions in persons with mNCD [8]. 

Against this backdrop, transcranial pulse stimulation (TPS), as described by Beisteiner et al. (2020) [9] and Beisteiner and Lozano (2020) [10], is a novel NIBS technique utilizing single, ultrashort ultrasound pulses (3 μs, with intervals of 200–300 ms). This method differs from other techniques like transcranial direct current stimulation (tDCS), which employ direct or induced electric currents, and transcranial magnetic stimulation (TMS) in its operational mechanics and delivery. TPS avoids potential NIBS constraints related to conductivity [11] and delivers stimulation capable of reaching deep brain regions [12]. This technique offers benefits over traditional focused ultrasound (tFUS) as the former minimizes tissue heating and the formation of standing waves [13,14,15]. This is achieved by employing very brief pulses that do not include periodic waves or extended sonication sequences. Standing waves can lead to unintended secondary stimulation peaks, which could reduce the spatial precision of tFUS. Additionally, TPS is distinguished as being the first ultrasound-driven NIBS method to receive clinical approval (CE mark), and has demonstrated its capacity to modulate the function in both stimulated and higher integrative brain areas [16]. The fundamental process behind TPS involves mechanotransduction, in which ultrashort ultrasound pulses stimulate the proliferation and differentiation of cultured neural stem cells. This activity is vital for the restoration of brain functions in diseases related to the central nervous system [17]. TPS may affect neurons and induce neuroplastic changes through several mechanisms, including enhancement of cell permeability [17], stimulation of mechanosensitive ion channels [18], and the release of nitric oxide, leading to vasodilation, increased metabolic activity, and angiogenesis [19].

Early evidence for the effectiveness and feasibility of TPS has emerged from studies on patients with unresponsive wakefulness syndrome [20] and Alzheimer’s disease [9,21], as well as those on healthy older adults [16]. An open-label study of 35 older adults with dementia undergoing a six-session TPS lasting for two weeks showed significant improvements in cognitive and depressive symptoms, which lasted up to three months [22]. Existing NIBS techniques, such as tDCS and TMS, have garnered substantial evidence supporting their efficacy in enhancing cognitive functions in mNCD. Research shows that tDCS can significantly improve general cognition, memory, and learning, although its impact on executive functions in mild NCD remains limited [23]. Similarly, TMS has demonstrated potential in boosting memory capabilities in elderly patients diagnosed with mild cognitive impairment (MCI), a condition similar to mNCD [24,25]. Building upon this foundational evidence, TPS emerges as a promising NIBS technique, particularly for its application in early-stage cognitive decline. Initial studies on TPS in patients with mild-to-moderate Alzheimer’s disease—a condition that precedes mNCD—have shown significant cognitive improvements, with patients reporting enhanced assessment scores and reduced symptom severity, all with minimal adverse effects [26]. Given the safety profile and cognitive benefits observed with TPS, alongside the established benefits of other neuromodulation techniques, there is a compelling basis to explore the use of TPS in an open-label study targeting older adults with mNCD. To date, no known TPS trials have specifically targeted older adults with mild NCD, a critical intervention period before the progression of cognitive degradation which may lead to irreversible impairment. By integrating TPS into the spectrum of NIBS techniques in mNCD, we aim to pave the way for its broader application as a viable adjunct treatment for cognitive rehabilitation and offer a new avenue for mitigating the impact of NCD at its onset.

While TPS has been shown to significantly improve cognitive performance in patients with mild to severe Alzheimer’s disease, as measured by various neuropsychological tests such as the Alzheimer’s Disease Assessment Scale (ADAS) and the Mini-Mental Status Examination (MMSE) [26,27], only one study has evaluated fMRI alongside cognitive assessments. It was observed that there were both increases and decreases in functional connectivity within specific brain regions [27]. The neuroimaging literature shows that mNCD is associated with abnormalities in functional connectivity, particularly in the default mode network (DMN) and other neurocognitive networks [28,29,30], as well as with reductions in cortical thickness [31]. Based on this evidence, we propose that TPS may enhance cognitive performance by modifying functional connectivity, leading us to focus on the DMN as our primary region of interest.

Addressing these research gaps, our study aimed to conduct an open-label trial to evaluate the effectiveness and tolerability of a two-week six-session TPS as to cognition and associated fMRI brain changes in older adults with mild NCD. We will analyze functional connectivity and cortical thickness of the brain to better understand the mechanistic underpinnings of TPS-induced cognitive improvements, offering a deeper understanding of its potential benefits. This research is pivotal as it not only explores a novel, non-invasive treatment option that could enhance cognitive function and quality of life but also sets the groundwork for future policies and clinical practices. By demonstrating the feasibility and efficacy of TPS, this study contributes significantly to the evolving landscape of interventions aimed at managing and potentially mitigating the progression of mild NCD in older adults.

## 2. Materials and Methods

### 2.1. Participants 

A total of 19 older adults with mild NCD participated in the 2022 study (12 females and 7 males, mean age = 74.32, SD = 6.65). Patients were directed to the study by psychiatrists from a specialist outpatient clinic within the public sector. They all satisfied the criteria for mild NCD as defined by the 5th Edition of the *Diagnostic and Statistical Manual of Mental Disorders* (DSM-5) at the time of their entry into the study, a diagnosis confirmed by at least one psychiatrist. All participants were 60 years of age or older and of Chinese descent. Exclusion criteria included an HK-MoCA score below the second percentile (adjusted for age and educational background), dependence on alcohol or drugs, the presence of unstable major medical or significant neurological conditions (such as brain tumors or aneurysms), hemophilia or other clotting disorders, thrombosis, substantial communication difficulties, and having any metal implants in the brain or the treated area of the head. Further all patients had stable anti-dementia therapy for at least three months prior to enrollment.

Informed consent was obtained from each qualified participant. The study was authorized by the Institutional Review Board of the University of Hong Kong and the Hospital Authority Hong Kong West Cluster (HKU/HA HKW IRB) (UW20-024). It was registered at ClinicalTrials.gov (ClinicalTrials.gov identifier NCT05331560). The study was conducted in accordance with the Good Clinical Practice standards and the Declaration of Helsinki. 

### 2.2. Study Design

**Figure 1:**
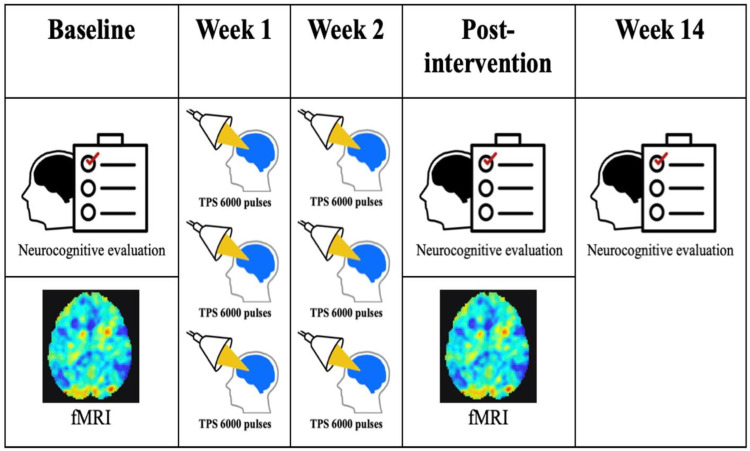
Transcranial pulse stimulation (TPS) intervention, functional magnetic resonance imaging (fMRI), and neurocognitive evaluation over 14 weeks. Each subject received three TPS sessions per week over two weeks.

This study was a two-week open-label trial of TPS. Based on the evidence that a major clinical advantage of TPS is its potential to act as an independent add-on therapy, patients received TPS as an ongoing and optimized standard clinical treatment [9]. Participants had assessments at baseline and post-intervention. After the baseline assessment, they received a total of six session TPS (6000 pulse each) as the intervention for two weeks (three sessions per week). Neurocognitive performance was evaluated at baseline and post-intervention (Figure 1). 

### 2.3. TPS

The intervention took place at the University of Hong Kong and was administered by trained medical staff. The TPS equipment included a portable transducer and an infrared camera system for MRI-based neuro-navigation (NEUROLITH, Storz Medical AG, Tägerwilen, Switzerland). TPS operates by emitting single ultrashort ultrasound pulses (3 µs) at energy levels typically ranging from 0.2 to 0.25 mJ/mm^2^ and pulse frequencies between 4 and 5 Hz [9]. A comprehensive brain stimulation strategy was used, distributing a total of 6000 TPS pulses evenly across all accessible brain regions per session. The stimulation was manually adjusted using different standoffs on the handpiece to control depth, and the handpiece was manually moved across the skull. Each session was recorded to allow for subsequent assessment of the pulse locations within the brain. 

### 2.4. Measures 

Participants underwent a semi-structured psychiatric diagnostic evaluation using the validated Chinese-bilingual edition of the Structured Clinical Interview for DSM Mental Disorders [32]. Basic elements of demographic information such as age, gender, education level, birthplace, marital status, number of children, financial status, family history of affective disorder, and household income were gathered. Their medical comorbidities were evaluated using the Cumulative Illness Rating Scale (CIRS) [33], and handedness was determined with the Edinburgh Handedness Inventory—Short Form [34]. Details regarding participants’ psychiatric history, including the onset age of cognitive symptoms and current medications and dosages, were documented at the start of the study. Medical histories and treatments were verified through patients’ electronic health records.

The core neurocognitive evaluation involved the Hong Kong Chinese version of the Montreal Cognitive Assessment (HK-MoCA) [35,36], conducted by a trained clinical investigator both at baseline and immediately after the two-week intervention period. This assessment, which is commonly used to evaluate overall cognitive function, has a scoring range from 0 to 30, with higher scores indicating better cognitive performance. Adverse effects of TPS were monitored in each session using a checklist that included potential symptoms like headache, pain or pressure, and mood changes. Secondary outcomes measured specific cognitive abilities such as attention, working memory, and executive functions, assessed through the forward and backward digit span (DS) [37], Stroop test [38], category verbal fluency test (VFT), and Trail Making Test (TMT) Parts A and B [39]. Daily functioning was evaluated using the Hong Kong Chinese version of the Lawton Instrumental Activities of Daily Living Scale (Chinese IADL) [40]. Additionally, the presence of depressive symptoms was assessed using the Hamilton Depression Rating Scale (HAMD-17) [41] and the Apathy Evaluation Scale (AES-C) was used for apathy symptoms.

### 2.5. Imaging Data Acquisition and Preprocessing

Imaging data for each participant used in the present study were collected at baseline and post-TPS treatment sessions at the University of Hong Kong’s MRI Research Centre on a Philips Achieva 3.0T scanner. Structural images were acquired with a 3D T1-weighted sequence (TR/TE/TI/Flip Angle 1820/3.75/1100 ms/7o). Resting-state fMRI were obtained using a T2*-weighted EPI sequence with the following parameters: TR = 2 s, TE = 32 ms; 32 slices; and repeated 150 times to provide time-series images. Spatial resolution was set to 1 × 1 × 1 mm voxel for T1-weighted images, and 3 × 3 × 4 mm for the resting-state fMRI images. During scanning, all participants were asked to keep still with their eyes open and refrain from engaging in systematic thoughts. 

Functional imaging data preprocessing was carried out using the CONN toolbox v19 [42]. Pre-processing steps comprised realignment, unwarping (subject motion estimation and correction), translation, slice–time correction, outlier detection (ART-based scrubbing), structural segmentation, and normalization. Following the guidelines for fMRI group comparisons outlined by Mikl et al. (2008) [43], images were smoothed using an 8 mm kernel at full-width at half maximum, considering that the voxel size, including gap, was 3.75 mm. The data underwent denoising through a band-pass filter ranging from 0.008 to 0.09 Hz. This included accounting for motion artifacts using six motion parameters and their first derivatives, incorporating five PCA components from cerebrospinal fluid and white matter masks (aCompCor) as described by Behzadi et al. (2007) [44], and implementing scrubbing for artifact removal. In the initial level of analysis, bivariate correlations were computed for the corrected time series across all voxels. Analysis focused on designated regions of interest (ROIs) such as the hippocampus, parahippocampus, and the default mode network, which were defined as nodes in an undirected graph, with connections above a certain threshold serving as edges. The ROIs were sourced from the Harvard–Oxford atlas. The networks under study included predefined networks from the CONN-Toolbox, derived from ICA analyses of the Human Connectome Project (HCP) dataset, which included 497 subjects and pertained to cognitive functions; a memory network based on a selection of regions identified in the literature; and a network involving the stimulated areas. The default mode network identified in the CONN analysis included the medial prefrontal cortex, bilateral lateral parietal cortex, and precuneus.

### 2.6. Functional Connectivity Analysis

Second-level seed-based analyses were conducted to assess the effect of local neural activity changes on whole brain functional connectivity (FC). The area comprising the hippocampus, parahippocampus, and default mode network was defined as the seed or region of interest for the FC analysis. Paired *t*-tests between the baseline and post-stimulation functional connectivity (FC) were calculated (correlation of the time series in the ROIs, 0.05 FDR seed-level correction, two-sided) on a group level. Seed regions for the hippocampus, parahippocampus, and the default mode network were defined as 6 mm spheres around the MNI coordinates from [45,46]. First, temporal correlations between the average fMRI signal values across the a priori seed regions and the time-course of every brain voxel were calculated and then normalized using the Fisher *z*-transformation. These connectivity *z*-maps were then entered into group-level regression models with values for mean frame-displacement (FD) of head motion, and treatment protocol as nuisance covariates. For each voxel within a seed, time-series data were collected from a spherical region with a radius of 5 mm and then averaged across all voxels within that seed to obtain the mean time series for the seed region. Subsequently, Pearson’s correlation analysis was conducted between the mean time series of each seed region and the time series of every voxel across the entire brain. This produced a map of correlation coefficients, which were subsequently converted to z-scores using the Fisher r-to-z transformation to enhance normality. These transformed scores were referred to as z-FC maps. The cluster-level threshold was set at *p* < 0.05 using family-wise error rate correction for multiple comparisons, with a voxel-wise threshold *p* < 0.001. Bonferroni-correction was applied to the α value to correct for multiple comparisons of the two a priori seeds tested, and areas with a minimum cluster size of 82 contiguous active voxels were identified as significant regions. The study aimed to investigate changes in functional connectivity pre- and post-transcranial pulse stimulation in late-life depression patients, focusing on specific ROIs, including the hippocampus, parahippocampus, and the default mode network. 

### 2.7. Cortical Thickness Analysis 

Prior to analysis, all images were screened for significant head motion. We employed a surface-based morphometry method for analyzing our anatomical images, utilizing the FreeSurfer software developed by Massachusetts General Hospital, Harvard University, Cambridge, MA, USA. This method includes a standardized pre-processing protocol to generate cortical surface models (meshes), followed by a longitudinal process to compute cortical thickness measurements at both examined time points [47,48,49]. We used FreeSurfer version 7.2, available at http://surfer.nmr.mgh.harvard.edu (accessed on 25 August 2024), to estimate cortical thickness by reconstructing the interfaces of grey/white matter and the cortical surface, and then measuring the distance between these surfaces at multiple points (vertices) across the cortical mantle [50,51]. Any errors in FreeSurfer’s initial Talairach alignments were identified through visual assessment of all images and corrected prior to the reconstruction of the cortical surfaces. Manual adjustments were made to rectify topological errors in the automated grey/white matter boundary delineation. Cortical thickness data for 68 regions based on the Desikan–Killiany atlas were derived from FreeSurfer [52]. All analyses were conducted blind to the identities of the subjects involved. For the group analysis of cortical thickness, a straightforward two-stage model was employed which calculates a linear fit for each subject separately to reduce the repeated measures to a single slope, followed by a regular cross-sectional analysis across subjects to detect any pre–post treatment effects. The mean differences in cortical thickness would be further analyzed with the psychological instruments for significant correlations, with the false discovery rate—(FDR)-corrected threshold of *p* < 0.05—used to correct for multiple comparisons. 

## 3. Results

Our study of TPS in participants with mNCD revealed specific functional connectivity changes and cognitive improvements. The fMRI results demonstrated an altered connectivity within key limbic structures, particularly around the left hippocampus and the right parahippocampus, which are crucial for memory processing and emotional regulation. These changes correlated with enhanced global cognitive performance as measured by the HK-MoCA, indicating that attenuated connectivity in these regions may be beneficial. However, no significant changes were observed in cortical thickness across all brain regions post-TPS, though an inverse correlation was found between the cortical thickness in the right precuneus and cognitive performance. These findings suggest that while TPS does not alter cortical thickness, it modulates functional connectivity in specific brain areas, potentially offering a non-invasive therapeutic approach which can be used to mitigate cognitive decline in mild NCD.

**Figure 2:**
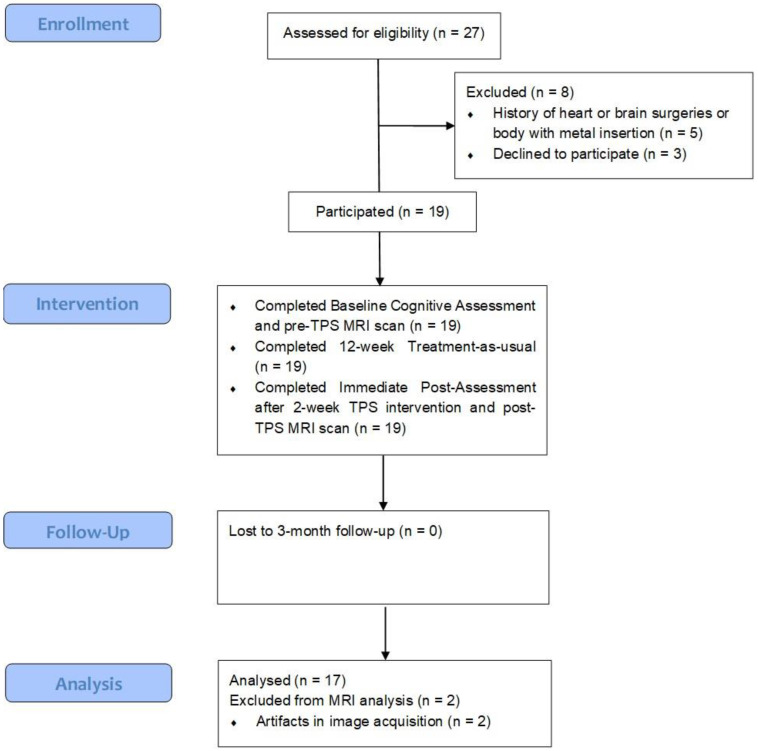
Study flowchart.

Sociodemographic characteristics of the participants are shown in Table 1. A total of 10 females and 7 males completed both pre- and post-MRI scan after TPS interventions (Figure 2). Their mean age was 74.06 years old. Slightly more than half were married (52.9%). Only two participants (11.8%) obtained a bachelor’s degree. Around a quarter (23.5%) of participants had a family history of mental disorders. No demographic characteristics were identified as significant covariates in association with the HK-MoCA scores. Participants were not restricted from food or water intake relative to the procedure, and no pain or discomfort was reported throughout the procedure. 

### 3.1. Functional MRI Results

**Figure 3:**
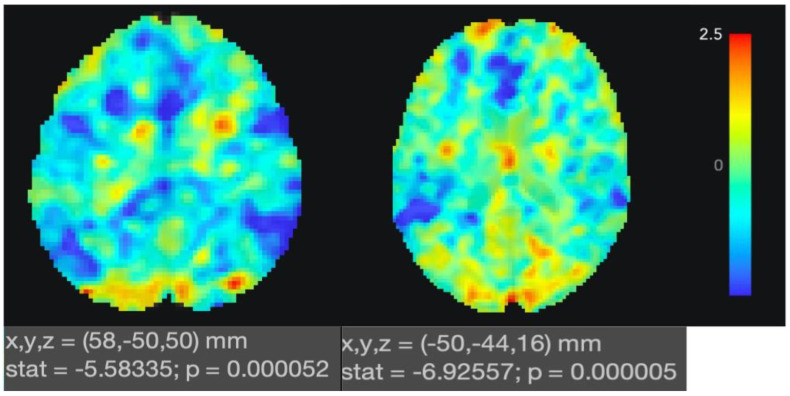
**Left side**: Differences in FC for the left hippocampus between the baseline and post-TPS treatment (paired *t*-test, *p* = 0.000052, multiple comparison correction, cluster size ≥ 85 voxels). The blue area represents the regions that have decreased FC. **Right side**: Differences in FC for the right parahippocampus between the baseline and post-TPS treatment (paired *t*-test, *p* = 0.000001, multiple comparison correction, cluster size ≥ 86 voxels). The blue area represents the regions that have decreased FC.

The fMRI investigations (*n* = 17) confirmed a specific down-regulation after TPS of the key structures within the brain’s limbic system that are involved in memory processing and emotional regulation. Paired *t*-tests between baseline and post-TPS functional connectivity (FC) with second-level seed-based analyses were conducted to assess changes pre- and post-TPS of FC for ROI of the left hippocampus and posterior right parahippocampus. The fMRI FC finding of the left hippocampus involved 85 voxels covering the left posterior division of the supramarginal gyrus (*t*-value: −5.58, size p-FDR: 0.030, size p-unc: 0.001393, size put: 0.001393) (Table 2 and Figure 3, left panel). For the ROI of the posterior right parahippocampus, a significant change in functional connectivity was observed, involving 86 voxels covering the atlas of the right posterior division of the supramarginal gyrus (*t*-value: −7.97, size PWE: 0.036482, size p-FDR: 0.029918, size p-unc: 0.000008) (Table 2 and Figure 3, right panel). The cluster’s significance is supported by FWE and FDR corrected *p*-values, indicating that the finding is unlikely to be a false positive, due to multiple comparisons.

Further, fMRI FC findings were compared to the neuropsychological scores retrieved from participants after the TPS intervention (Table 3). Multiple linear regression revealed that the neuropsychological post scores for the HK-MoCA totals were associated with the post-TPS seed-based FC of the posterior left parahippocampus, at *B* = 0.087 (CI, 0.007–0.167; *p* = 0.038). The decrease in post-TPS FC of the posterior left parahippocampus predicted higher global cognition. There was no significant correlation between the neuropsychological scores and the FC values of the posterior right parahippocampus region. 

### 3.2. Cortical Thickness Results

The mean cortical thicknesses of all brain regions for participants before and after the TPS intervention (*n* = 17) were analyzed. Analysis revealed no significant difference in cortical thickness. By comparing the mean difference of cortical thickness with the neuropsychological measurements, HK-MoCA scores showed a significant inverse correlation (r = −0.764, *p* = 0.019) with the right precuneus after FDR-correction (Table 4). A thinner cortex in right precuneus was associated with better performance on HK-MoCA scores. 

## 4. Discussion

This preliminary study is the first endeavor to explore the potential cognition-enhancing effects and fMRI changes associated with TPS in older adults with mild NCD. Specifically, our findings demonstrate that TPS attenuates functional connectivity (FC) in the posterior left parahippocampus, which correlates with improved global cognition (HK-MoCA scores). The significant findings in the seed-to-voxel analysis, after controlling for multiple comparisons (FDR), suggest a robust effect of the intervention on brain connectivity. These findings were further supported by cortical thickness analyses, which revealed an inverse correlation between cortical thickness in the right precuneus and cognitive performance, suggesting that even subtle neuroanatomical changes post-TPS can have functional implications. No change in cortical thickness was observed within this 2-week TPS protocol. TPS was safe and well-tolerated by older adults, as discussed in our previous work [53].

Our findings align with previous neuromodulation research. Similar to TMS and tDCS, TPS modulates functional connectivity of brain regions within a network targeted by NIBS [54,55]. Drawing parallels with a previous TPS study focusing on patients with Alzheimer’s diseas, Dörl et al. [21] targeted TPS stimulation on predominantly left dorsolateral prefrontal cortex, and demonstrated a reduced connectivity of the visuo-constructive network with enhanced cognitive performance. Subsequently, in a separate TPS study conducted on healthy participants, global efficiency significantly increased within the cortical sensorimotor network, indicating an upregulation of the stimulated functional brain network, but there was no difference in the performance of behavioral tasks [16]. Contrastingly, in our study, a global brain stimulation approach was associated with an observed decrease in FC in the left hippocampus and posterior right parahippocampus post-TPS, which correlated with higher global cognition in older adults with mild NCD. The hippocampal formation, including the parahippocampus, plays a pivotal role in memory processing and emotional regulation [56]; their overactivation or enhanced connectivity could potentially lead to inefficient processing or “neural noise” [57]. Hence, a downregulation or decreased functional connectivity in these regions might lead to cognitive improvements, as reflected in the MoCA scores. It is purported that our TPS global-stimulation approach might have broader effects in brain connectivity, affecting multiple brain areas and networks simultaneously [58]. 

Conversely, alterations in functional connectivity in the precuneus, a component of the default mode network (DMN)—active during cognitive rest—are common in NCD [59]. Our study reveals an intriguing inverse correlation between MoCA scores and the right precuneus’s cortical thickness. This observation suggests that the right precuneus could be a structural correlate of TPS response in mild NCD [60]. Correspondingly, previous research found a significant correlation between increased cortical thickness in DMN (specifically, the left superior parietal lobule and left precuneus) and enhanced cognitive performance among Alzheimer’s disease patients [61]. This implies that TPS can potentially increase cortical thickness, possibly offsetting the cortical atrophy seen in Alzheimer’s disease. 

However, our study found no significant difference in the mean cortical thickness across all brain regions in elderly patients with mild NCD following TPS. Previous research involving Alzheimer’s disease has found a significant correlation between increased cortical thickness in DMN (specifically, the left superior parietal lobule and left precuneus) [61]. It is plausible that our sample was anodyne, which was different from the study’s research focus on Alzheimer’s disease. Also, given the complexity and heterogeneity of brain networks, the impact of TPS on cortical thickness may be region-specific and not captured by a global measure [62]. Another consideration is the limited sample size of our study, which could have reduced the power of our analysis to detect significant changes in cortical thickness. On the other hand, we observed an inverse correlation between MoCA scores and the baseline right precuneus’s cortical thickness. It has been found that a thinner cortex before treatment is correlated with a more favorable rTMS response [60]. We postulate that thinner right precuneus for mild NCD might be a structural correlate of TPS response, but this remains to be verified in further studies.

Non-invasive stimulation procedures, such as those modulating theta interactions in the brain, have been shown to restore neural synchronization patterns and improve working memory in older adults. These improvements can last beyond the stimulation period [63]. There is abundant research in tDCS on enhancing cognitive functions, including working memory and motor performance, in older adults. Studies indicate significant improvements in cognitive tasks when tDCS is applied; the association with these cognitive effects has been limited to the targeting of the prefrontal cortex [64,65,66]. TPS is a global-stimulation technique that can also target the wider regions of the brain, leading to a more global improvement in neurocognitive performance and cognitive health. This indicates that integrating TPS into the spectrum of NIBS techniques in mNCD might develop a viable adjunct treatment for cognitive rehabilitation and a potential new approach for mitigating the impact of NCD at onset.

Furthermore, the effectiveness of TPS in older adults with mNCD may be significantly influenced by several tailored factors, which are specific to the application and mechanics of TPS. Utilizing priming protocols prior to TPS sessions can modify the neuromodulatory effects, potentially enhancing cognitive outcomes by altering corticospinal excitability (CSE) based on synaptic activity history [67]. The intensity and frequency of TPS need careful calibration as they can affect cerebral blood flow velocity (CBFv) and vasomotor reactivity, which are crucial for maintaining cognitive health [68]. Additionally, incorporating real-time monitoring of neuronal states through EEG during TPS sessions allows for adjustments in stimulation parameters, which can minimize variability and optimize results [69,70]. Spacing multiple TPS protocols within a session, similarly to other NIBS techniques, might enhance the reliability and prolong the effects of the treatment. This approach helps stabilize the after-effects and reduce variability in response [69]. Furthermore, tailoring TPS to interact with specific brain oscillatory activities could normalize abnormal network activity, thereby improving clinical outcomes in neurocognitive disorders [71]. Genetic factors, such as variations in the brain-derived neurotrophic factor (BDNF) gene, might also modulate the efficacy of TPS-induced neuroplasticity, suggesting a personalized approach to optimizing TPS protocols based on individual genetic profiles [72]. By focusing on these specific aspects, the therapeutic potential of TPS for cognitive enhancement in mild NCD could be significantly advanced in future research.

Our pilot study provides a perspective on TPS as an add-on treatment for older adults with mild NCD, encompassing comprehensive clinical evaluations and neurocognitive and neuroimaging assessments. However, the study also has limitations that must be acknowledged. The open-label design and the small sample size of only 17 participants limit the generalizability of the findings. Without a control group, it is challenging to definitively attribute the cognitive and neuroimaging changes to TPS alone, as placebo effects or natural fluctuations in cognitive performance could also be influencing factors. Additionally, the short duration of the intervention (two weeks) and follow-up may not adequately capture long-term effects and sustainability of cognitive improvements. Future studies should consider incorporating randomized controlled trials, larger sample sizes, and extended follow-up periods to build on these preliminary findings and address these limitations. In addition, acknowledging the visual representation of our findings, while effective for data conveyance, could be further refined by exploring graph-theoretical network visualization toolbox in future iterations of this research [73]. Furthermore, although direct analysis using the Neurosynth database was not feasible in our study, previous quantitative meta-analyses provide substantial support for the role of the hippocampal formation in memory and emotional processes [74,75]. 

On the theoretical front, our findings enrich the understanding of the neural mechanisms underlying cognitive enhancements following neuromodulation and underscore the need for further exploration into how TPS specifically affects brain connectivity and function. From a practical perspective, our study highlights significant clinical implications for the treatment of mild NCD. These results suggest that TPS could be a potentially effective non-invasive intervention used to mitigate cognitive decline. Combining neuromodulation with cognitive interventions or exercise may enhance treatment efficacy [76]. One trial has shown that the use of neuromodulation technique of ultrasound stimulation enhances the opening of the blood–brain barrier, which shows promise for promoting drug delivery more efficiently in neurocognitive disorders [77]. Clinically, the integration of TPS could offer a safer alternative or adjunctive treatment to pharmacological treatments, which often carry the risk of adverse effects. From a research perspective, this study sets a foundational basis for future investigations, encouraging larger, randomized trials to confirm these findings and explore the long-term effects and optimal parameters of TPS. Ultimately, this could lead to neuromodulation strategies which are more personalized and tailored to individual neurocognitive profiles, thereby enhancing the efficacy of interventions for cognitive impairments in older adults. Future studies could explore the optimal parameters for TPS intervention, such as the frequency, duration, and intensity of stimulation, as well as comparative effects of global- versus targeted-TPS stimulation on specific brain regions associated with cognition [78]. Furthermore, translational research on the mechanisms of action and effects on the cerebral network mechanism is warranted. It has been suggested that stimulation of mechanosensitive ion channels leads to elevated metabolism, angiogenesis, and anti-inflammatory responses due to nitric oxide release in the targeted regions. This stimulation influences the production of vascular growth factors (VEGF), enhances neurogenesis (eNGF and GF-2), and increases levels of brain-derived neurotrophic factors (BDNF) [9,79].

## 5. Conclusions

Our study provides a new exploratory insight into the effectiveness of TPS for enhancing neurocognitive functions in older adults with mNCD. In our study, we observe altered functional connectivity within the deep brain regions, specifically the posterior supramarginal gyrus, post-TPS, which may be associated with the neurocognitive improvement. This underscores the potential of TPS as a timely intervention during the critical window before cognitive decline becomes irreversible. The safety, feasibility, and preliminary positive outcomes of TPS observed in our study not only highlight its potential as a viable therapeutic option but also set the stage for extensive future research. Further research is necessary, particularly through randomized, blinded trials that involve comprehensive whole-brain evaluations and neurocorrelation analyses. Such studies will allow for a more thorough investigation into the changes associated with TPS and a deeper understanding of the underlying mechanism of this NIBS technique as adjunctive treatment in clinical settings. These efforts are essential for advancing theoretical knowledge and developing robust methodological strategies to effectively mitigate the progression of mild NCD.

## Figures and Tables

**Table 1:** Sociodemographic characteristics of TPS subjects involved in MRI analysis (*n* = 17).

	Overall (*n* = 17)		*^#^* χ^2^
	*n*	%	
Gender			0.49
Male	7	41.2	
Female	10	58.8	
Age			0.17
60–69	4	23.5	
70–79	9	53.0	
80 or above	4	23.5	
Marital status			0.67
Married	9	52.9	
Single/Separated/Widowed	8	47.1	
Education level			0.56
Elementary or below	8	47.1	
High School/College	7	41.2	
University or higher	2	11.7	
Family history of mental disorders			0.16
Yes	4	23.5	
No	13	76.5	

^#^ Chi-squares comparing HK-MoCA Baseline Scores.

**Table 2:** Significant differences in FC pre- and post-TPS treatments among the participants.

Brain Region	MNI Coordinates			Cluster Size	*t*-Value
	*x*	*y*	*z*		
Supramarginal gyrus (posterior right)	58	−50	50	85	−5.58
Supramarginal gyrus (posterior left)	−50	−46	16	86	−7.97

**Table 3:** Differences in correlation between FC values of supramarginal gyrus (posterior left) and neuropsychological tests among the participants.

Variable	*B*	95% CI for *B*	*p*-Value
(constant)	−3.664		
HK-MoCA Total	0.087	0.007, 0.167	0.038 *
VFT—60 s	0.033	−0.016, 0.083	0.146
Stroop Interference	−0.001	−0.016, 0.014	0.857
Forward DS Span Length	0.593	−0.077, 1.263	0.072
Forward DS Total	−0.253	−0.564, 0.058	0.090
Backward DS Span Length	0.207	−0.746, 1.159	0.601
Backward DS Total	−0.129	−0.653, 0.396	0.556
HAM-D Total	0.053	−0.017, 0.123	0.110
AES-C Total	0.005	−0.019, 0.029	0.592
Chinese IADL Total	−0.009	−0.072, 0.053	0.722

* *p* < 0.05. Model adjusted *R*^2^ 0.880. MoCA = Montreal Cognitive Assessment; VFT = Verbal Fluency Test; DS = Digit Span; HAM-D = Hamilton Depression Rating Scale; AES-C = Apathy Evaluation Scale-Clinician; IADL = Instrumental Activities of Daily Living; CI = Confidence Interval.

**Table 4:** Significant correlation found in mean differences between cortical thickness of right precuneus and HK-MoCA on participants with mild cognitive impairment (*n* = 17).

	Pre-Intervention	Post-Intervention	*r*	*p*-Value
	Mean (S.D.)	Mean (S.D.)		(FDR Adjusted)
HK-MoCA	19.41 (4.784)	20.76 (4.280)	−0.764	0.019 *
Right Precuneus	2.080 (0.179)	2.040 (0.176)		

r = Pearsons correlation chi-square; * *p*-value adjusted to false discovery rate (FDR)-corrected values (*p* < 0.05). S.D. = Standard Deviation.

## Data Availability

The original contributions presented in the study are included in the article, further inquiries can be directed to the corresponding author.
